# Leptospermum extract (QV0) suppresses pleural mesothelioma tumor growth *in vitro* and *in vivo* by mitochondrial dysfunction associated apoptosis

**DOI:** 10.3389/fonc.2023.1162027

**Published:** 2023-07-05

**Authors:** Huaikai Shi, Le Zhang, Ta-Kun Yu, Ling Zhuang, Helen Ke, Ben Johnson, Emma Rath, Kenneth Lee, Sonja Klebe, Steven Kao, Karl Lijun Qin, Hong Ngoc Thuy Pham, Quan Vuong, Yuen Yee Cheng

**Affiliations:** ^1^ Asbestos and Dust Diseases Research Institute, Sydney, NSW, Australia; ^2^ Institute for Biomedical Materials & Devices (IBMD), Faculty of Science, The University of Technology, Sydney, NSW, Australia; ^3^ Giannoulatou Laboratory, Victor Chang Cardiac Research Institute, Sydney, NSW, Australia; ^4^ Sydney Medical School, University of Sydney, Sydney, NSW, Australia; ^5^ Pathology, Concord Repatriation General Hospital, Sydney, NSW, Australia; ^6^ Pathology, Flinders Health and Medical Research Institute, Flinders University, Bedford Park, SA, Australia; ^7^ Department of Medical Oncology, Chris O’Brien Lifehouse, Sydney, NSW, Australia; ^8^ Quality Global Supply Pty. Ltd., Tuggerah, NSW, Australia; ^9^ College of Engineering, Science and the Environment, University of Newcastle, Sydney, NSW, Australia; ^10^ Faculty of Food Technology, Nha Trang University, Nha Trang, Vietnam

**Keywords:** *Leptospermum*, mitochondria dysfunction, apoptosis, pleural mesothelioma, anti-cancer (anticancer) drugs

## Abstract

Pleural mesothelioma (PM) is a highly aggressive, fast-growing asbestos-induced cancer with limited effective treatments. There has been interest in using naturally occurring anticancer agents derived from plant materials for the treatment of PM. However, it is unclear if an aqueous extract from *Leptospermum polygalifolium* (QV0) has activity against PM. Here we investigated the anti-cancer properties of QV0 and Defender^®^ (QV0 dietary formula) *in vitro* and *in vivo, respectively*. QV0 suppressed the growth of eight PM cell lines in a dose-dependent manner, effective at concentrations as low as 0.02% w/v (equivalent to 0.2 mg/ml). This response was found to be associated with inhibited cell migration, proliferation, and colony formation but without evident cell cycle alteration. We observed mitochondrial dysfunction post-QV0 treatment, as evidenced by significantly decreased basal and maximal oxygen consumption rates. Ten SCID mice were treated with 0.25 mg/g Defender^®^ daily and exhibited reduced tumor size over 30 days, which was associated with an average extension of seven days of mouse life. There was no evidence of liver toxicity or increased blood glucose post-treatment in animals treated with Defender^®^. Significantly enhanced tumor apoptosis was observed in the Defender^®^-treated animals, correlating to mitochondrial dysfunction. Lastly, the high levels of polyphenols and antioxidant properties of QV0 and Defender^®^ were detected in HPLC analysis. To the best of our knowledge, this study constitutes the first demonstration of an improved host survival (without adverse effects) response in a QV0-treated PM mouse model, associated with evident inhibition of PM cell growth and mitochondrial dysfunction-related enhancement of tumor apoptosis.

## Graphical Abstract

Illustration of *Leptospermum* extract suppressing pleural mesothelioma tumor growth *in vitro* and *in vivo* by mitochondrial dysfunction-associated apoptosis.
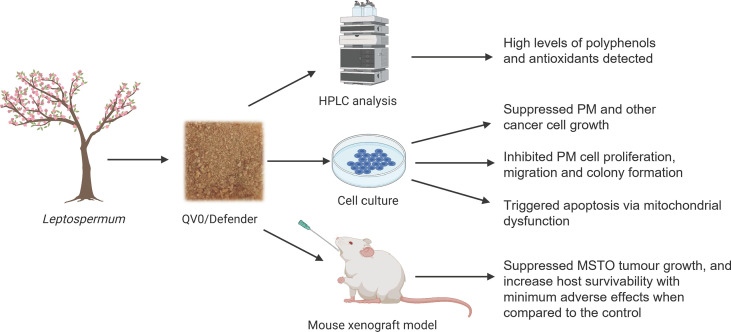

## Introduction

Pleural mesothelioma (PM) is an aggressive thoracic malignancy with a poor prognosis and a high symptom burden that is caused by previous exposure to asbestos. The current treatment options for PM patients include cisplatin and pemetrexed (1), with the possible addition of bevacizumab to chemotherapy (2), or combination immunotherapy with ipilimumab and nivolumab (3, 4). Despite recent advancements in PM treatment with the introduction of immunotherapy, the average survival time of PM patients remains poor, with a median survival of around 18 months (5). Novel treatment agents and approaches are desperately needed to improve PM patient survival outcomes.

Natural products, including plants, microbial products, and marine sources, have provided key substrates for the production and development of anti-cancer drugs for decades (6). There remains great interest in the potential for natural products to produce new anti-cancer therapies. Unlike the significant toxic side effects associated with standard chemotherapy- and immunotherapy-based cancer treatment options, novel drug candidates developed from natural products induce minimal toxicity in healthy non-malignant cells (7). A variety of natural substances have been tested *in vitro* and in PM animal models. These include various polyphenolic compounds such as curcumin (8), resveratrol (9), and quercetin (10), as well as extracts from plants such as artichoke leaf (*Cynara scolymus*) (11), olive leaf (*Olea europaea* L.) (12), *Glychyrrhiza inflata* (13), *Filipendula vulgaris* (14), and microbial products such as Maunomycin A (15) and JBIR-23 (16).

Manuka (*Leptospermum* sp.) has demonstrated apoptotic (17), immunomodulatory, and antiproliferative effects in breast, colorectal, and melanoma tumor cell lines (18), and in animal models (19). Although the mechanism for anti-tumor activity is yet to be fully elucidated, there is evidence to indicate that manuka induces apoptosis through alteration in aquaporin-3 signaling, increasing intracellular reactive oxygen species, and disruption of intracellular calcium homeostasis, leading to cell death (17). The mitochondria, a key regulator of cellular physiological processes such as cellular respiration, apoptosis, DNA repair, and cell cycle control, have been implicated in various malignancies, including pleural mesothelioma. Abnormal mitochondrial function and oxygen consumption are associated with the accelerated growth and progression of mesothelioma (20). Therefore, understanding the involvement of mitochondrial responses to *Leptospermum* treatment is needed.

The present study represents the first report on the anticancer activity of a specific extract from the manuka honey tree, *Leptospermum polygalifolium* (QV0, P116949.AU), in PM. Cultured PM cells treated with QV0 exhibited reduced proliferation, migration, and impediments to colony formation and mitochondrial function. Furthermore, QV0 delivered as a dietary supplement using manuka honey as a base (Defender^®^) in animals demonstrated tumor-suppressive activity without biochemical or anatomical evidence of toxicity. These findings provide a rationale for prospective translational research aimed at facilitating the clinical implementation of a QV0-based PM treatment.

## Materials and methods

*L. polygalifolium* extract (QV0, P116949.AU) and dietary supplement (Defender^®^) were supplied by Quality Global Supply Australia Pty. Ltd., Tuggerah, NSW, which produces natural supplements and honey-based products. QV0 was prepared from *L. polygalifolium* leaves and small stems using aqueous extraction, followed by spray drying to obtain powdered extract. For feeding animals, QV0 was prepared in the form of a dietary supplement (Defender^®^), which includes 5% of QV0, 15% of citrus pomace powder, and 80% of honey. In the present study, 5 g of QV0 or Defender^®^ was dissolved in 100 ml of warm PBS to make a 5% stock solution for *in vitro* and *in vivo* experiments.

### *In vitro* studies

#### Cell lines and maintenance

Five human PM cell lines (H28, H2052, H2452, H226, and MSTO) and the immortalized mesothelial cell line, MeT-5A were obtained from the American Type Culture Collection (ATCC, Manassas, VA, USA). The primary mesothelioma cell line, MM05 (21), was generated at the University of Queensland Thoracic Research Centre (The Prince Charles Hospital, Brisbane). Ren cells (22) were provided by Laura Moro of the University of Piemonte Orientale A. Avogadro, Novara, Italy. VMC40 cells (23) were provided by Michael Grusch from the Institute of Cancer Research, Department of Medicine, Medical University of Vienna. Mouse mesothelioma cell line (AC29) was purchased from Cell Bank Australia. All other primary mesothelioma cells were established in the Asbestos Disease Research Institute (ADRI) laboratory. Cells were cultured at 5% CO_2,_ 37°C, and 95% humidity in RPMI 1640 with 10% fetal bovine serum (FBS). All media and FBS were obtained from Life Technologies (Carlsbad, CA, USA).

#### Cell proliferation assay

Briefly, 2,500 cells were seeded in 96-well culture plates in 100 μl medium per well overnight. Cells were treated with 50 μl QV0 (IC_50_) for 72 h, followed by the subsequent addition of 15 μl Alamarblue^®^ (50 ml PBS containing 0.075 g Resazurin, 0.0125 g Methylene Blue, 0.1655 g Potassium hexacyanoferrate (III), and 0.211 g Potassium hexacyanoferrate (II) trihydrate, filter-sterilized, and stored at 4°C in the dark). The cells were then incubated for 4 h at 37°C as described (24). Fluorescence intensity was measured at 590 nm with 544 nm excitation using a FLUOstar Optima (BMG LabTech, Ortenberg, Germany). Fluorescence intensity was calculated as a percentage of the total intensity of the untreated control cells. Experiments were performed three times with three replicates each time, except for experiments involving slow-growing non-cancer primary fibroblasts that were performed four times with two or three replicates.

#### Cell migration assay

Cell migration of various cell lines was measured using a scratch (wound-healing) assay. Briefly, cells were plated in 24-well plates, and at 24 h post-seeding, 10 µg/ml camptothecin (Sigma-Aldrich) was added to stop cell proliferation; at the same time, a cross-shaped scratch was made using a 200 µl plastic pipette tip, and QV0 (IC_25_) was added. At 12 and 24 h post-scratch, microscopic imaging was carried out with a ×20 objective (Leica DMi1). Each experiment group performed it in duplicate.

#### Clonogenic assay

Cells of each cell line were seeded in 6-well culture plates at a seeding density of 2,500 cells/well. QV0 (IC_25_) was added at 2 h post-seeding, and culture plates were incubated for 10–14 days at 37°C. Cells were then fixed with 70% ethanol and stained with 0.1% crystal violet before being photographed for colony counting using a ZEISS Stemi508 microscope.

#### Live cell image

Mesothelioma cells (H2052, H28) with or without treatment with QV0 were studied using an Olympus Ti microscope with a time series setup. Briefly, 2,500 cells were seeded on a glass-bottomed 96-well plate, and after an overnight incubation, cells were treated with control (medium only) or QV0. Cells were immediately analyzed on an Olympus Ti microscope, with images taken every 30 min. Images were taken over 24 h and converted to movies.

#### Cell cycle analysis

Mesothelioma cells were treated with QV0 (IC_25_), and at 48 h post-treatment, the cells were harvested and washed three times with phosphate-buffered saline (PBS). The cells were subsequently fixed in 70% ethanol for at least 30 min. For cell cycle analysis, the fixing solution was removed, and cells were treated with 0.01% RNase (10 mg/ml, Sigma-Aldrich) and 0.05% propidium iodide (PI) (Sigma-Aldrich) in PBS for 30 min at 37°C in the dark. The cell cycle distribution was determined on a CytoFLEX flow cytometer (Beckman Coulter, Miami Lakes, FL) within 30 min. The flow cytometer was calibrated using calibration beads according to the manufacturer’s instructions (CytoFLEX, Beckman). The flow cytometer was routinely operated at the slow flow rate setting (μl sample/minute), and the data acquisition for a single sample typically took 3–5 min. For each sample, 10,000 events of single cells were counted, and the cell cycle was analyzed using FlowJo software (Ashland, OR, USA).

#### Seahorse extracellular flux analysis

The Seahorse XF24 Extracellular Flux Analyzer (Agilent, CA, USA) was used to measure the respiration activity of mesothelioma cells. Cells were seeded at a seeding density of 8 ×  10^4^ cells per well in an XF24 plate overnight and treated with and without QV0 (IC_25_) for 24 h. The mitochondrial stress test was performed according to the manufacturer’s instructions. Briefly, 1 μM oligomycin (oligo), 0.3 μM FCCP, and 1 μM rotenone and antimycin A were added, and the relative levels of basal, maximal respiration, and reserved mitochondrial capacity were calculated based on OCR data obtained from the Mito stress tests using Seahorse Wave software for XF analyzers (Agilent, CA, USA).

### *In vivo* studies

#### PM xenograft mouse model

To study the *in vivo* response of QV0, a food formula called ‘Defender^®^’ (consisting of honey as a base and containing QV0) was used as a supplement to feed the animals. A total of twenty SCID mice (8-week-old females) were intraperitoneally (i.p.) injected with 1 × 10^6^ (in 200 μl medium) human mesothelioma cells (MSTO-211H) pre-transfected with a stable pGL4-51lu luciferase construct for visualization of tumor growth. Mice carrying MSTO-pGL4-51lu tumors were i.p. injected with 200 μl of luciferin (150 mg/kg) for tumor visualization. The tumor emits a visual light signal that can be measured by IVIS (PerkinElmer, Waltham, USA). A tumor was considered ‘tumor-bearing’ once it had grown to 3–4 mm and tumor nodules were visualized. Animals were then evenly separated into two groups (control and Defender^®^). In the Defender^®^ group, animals were treated with 5 mg/mouse/20 g body weight of Defender^®^ in 200 µl volume as a daily supplement, which was administered orally using an oral gavage. Mice were monitored and sacrificed in accordance with SHLD animal ethics (2017/021).

#### Histological assessment

The harvested animal tumor, liver, spleen, and stomach tissues were embedded in paraffin. Multiple 4-μm sections were stained with hematoxylin and eosin (H&E) (Sigma-Aldrich) for general histological analysis by two pathologists.

#### TUNEL assay

An *in situ* cell death detection kit (Roche, Basel, Switzerland) was used for the detection and quantification of tumor cell apoptosis. Briefly, formalin-fixed tumor tissue sections were dewaxed according to standard procedures and then incubated in 0.1M citrate buffer PH 6.0 at 70°C for 1 h. The slides were blocked with Tris–HCL, 0.1 M PH 7.5, containing 3% BSA and 20% normal bovine serum for 30 min at room temperature, followed by the addition of 50 ul of TUNEL reaction mixture to the slides and subsequent incubation for 60 min at 37°C in a humidified atmosphere in the dark.

#### Liver toxicity test

Liver toxicity was assessed by aspartate aminotransferase (AST) and alanine aminotransferase (ALT) serum concentrations using commercial assays (MAK055 and MAK052, Sigma-Aldrich). All experimental work was performed as per the kit instructions.

#### Statistical analysis

For the proliferation assays, the QV0 IC50 concentration at which 50% of cells were viable was calculated by modeling cell response to QV0 treatment using a sigmoid function (25) as described previously (24). Briefly, the sigmoid function used to predict cell proliferation, *y*, was:


$$y=A+\left(B-A\right)*\frac{1}{\left(1+\exp\left(\frac{\left(xmid-x\right)}{scale}\right)\right)}$$

where A is the left asymptote (cell response at QV0 treatment concentration of 0), B is the right asymptote (cell response at highest QV0 treatment concentration), xmid is the transition point (IC50) of the cells treated with QV0, scale is an x-axis scale parameter impacting the slope of the transition, and x is log10 of the QV0 treatment concentration (thus rendering the curve symmetrical and suitable for modeling using log-likelihood). The best-fitting parameters for a given model were determined by the maximum log likelihood method using the optimx package (26) in R (27). The IC50 (concentration at which 50% of cells are viable) was calculated as the sigmoidal transition point resulting from the model having the best-fitting parameters. The IC50 standard deviation was calculated as the standard deviation of the transition points for each experiment, modeled individually as a sigmoid function. The cell cycle profile of a cell line after QV0 treatment was compared to that of the same cell line without QV0 treatment in the following way. The cell cycle profile is the percentage of cells in each cell cycle phase. The differences between the QV0-treated and non-treated cells were calculated. A Student’s t-test in R (27) was used to determine whether the differences were zero. ANOVA and paired t-tests were also used in this study, with significance set at P<0.05.

### Chemical composition studies of QV0 and Defender^®^

#### Analysis of total phenolic content

The level of TPC was determined by the Folin–Ciocalteu method (AOCS, 1990), modified for the microplate. Water was used as a blank, and gallic acid was used as the standard for a calibration curve. Approximately 15 μl of sample, standard, or blank was placed into 24-well microplates, followed by the addition of 240 μl of diluted Folin–Ciocalteu (FC) phenol reagent (6.25%). The mixture was incubated in the dark for 10 min at room temperature, followed by the addition of 15 μl of 20% sodium carbonate. The mixture was then incubated for a further 20 min in the dark, and the absorbance was measured at 765 nm using a FLUOstar Optima microplate reader (BMG LabTech, Ortenberg, Germany). The TPC was interpolated from the calibration curve and expressed in milligrams of gallic acid equivalent per gram of sample (mgGAE/g).

#### Ferric reducing antioxidant power

The antioxidant activity of the sample was evaluated using the ferric reducing antioxidant power assay (FRAP) according to the previously published method with modifications to the microplate. Briefly, a FRAP working solution was prepared freshly by mixing three reagents: (A) 300 mM acetate buffer (PH 3.6), (B) 10 mM 2,4,6 tripyridyl-s-triazine (TPTZ) in 40 mM HCL solution, and (C) 20 mM ferric chloride. Trolox was used as the standard for a calibration curve. Approximately 50 ul of the sample, blank, or standard was placed into 24-well microplates, followed by the addition of 300 ul of FRAP working solution. The mixture was incubated for 30 min at room temperature, and the absorbance was measured at 593 nm using a FLUOstar Optima microplate reader (BMG LabTech, Ortenberg, Germany). The antioxidant activity of plasma was expressed in milligrams of Trolox equivalent per gram of extract (mgTE/g).

#### Scanning for major phytochemicals

Major phytochemicals in *Leptospermum* extract were determined using a Shimadzu HPLC system (Shimadzu, Japan) fitted with a reverse phase column (Luna 5u Phenyl-Hexyl 250 × 3.00 mm 5 um) (Phenomenex) maintained at 35°C in a column oven (CTO-20A, Shimadzu) with a photodiode array detector (SPD-M40). The mobile phase consisted of 0.1% formic acid (Solvent A) and absolute acetonitrile (Solvent B). An auto injector (SIL-20A) was used to inject 25 µl sample volumes onto the HPLC at a flow rate of 0.7 ml/min with a gradient elution schedule as follows: 0–10 min, 0% B; 10–45 min, 40% B; 45–60 min, 60% B; 60–70 min, 60% B; 70–80 min, 0% B; and 80–85 min, 0% B. Kaempferol was used as the standard for quantification. Quercetin was used as the standard for a calibration curve for quantification of quercetin and two unknown phytochemicals, and the results were expressed as micrograms of quercetin per gram of sample (μgQE/g).

## Results

**Figure 1:**
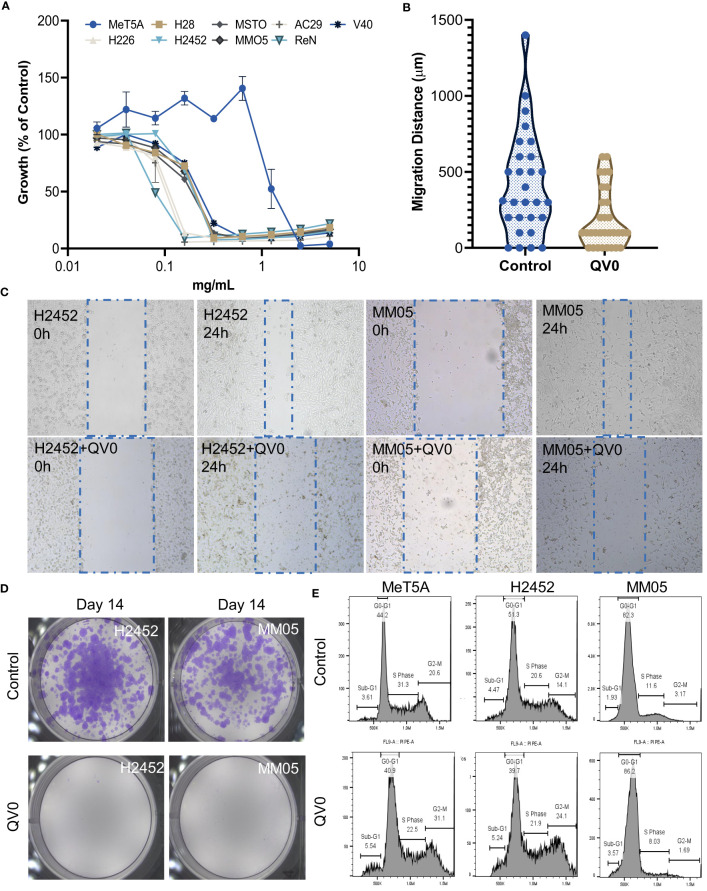
**(A)** QV0 suppressed the growth of all tested PM cell lines but not the immortalized mesothelial cell, MeT5A. The fluorescence intensity of the QV0-treated cells is presented as a percentage of the intensity of the untreated control cells. All cells were treated with QV0 (0.01 mg/ml to 10 mg/ml) for 72 h. **(B)** QV0 significantly inhibited the tumor cell migration distance compared to the untreated control cells. Blue dots indicate the migration distance without QV0 treatment, and yellow dots indicate the migration distance after QV0 treatment (IC25) for 24 h. **(C)** Representative images showing an inhibition of cancer cell (H2452, MM05) migration following QV0 treatment. **(D)** Representative images showing an evident suppression of colony formation in cancer cell lines H2452 and MM05 following 14 days of QV0 treatment (IC25) with respect to the untreated control cells. **(E)** Representative images depicting unaltered cell cycle profiles of immortalized mesothelial (MeT5A) and mesothelioma cancer (H2452 and MM05) cells at 48 h post-QV0 treatment (IC25). For each sample, 10,000 events of single cells were counted, and the cell cycle phases were subsequently analyzed using FlowJo software. N = 3 per cell line.

We applied functional *in vitro* assays to understand the anti-cancer effects of QV0. The Alamarblue^®^ cell proliferation assay was used to assess the anti-proliferative effect of QV0 on 8 pleural mesothelioma (PM) cell lines and one immortalized mesothelial (MeT5A) cell(s). Results indicated that QV0 suppressed the growth of all eight of the tested PM cell lines in a dose-dependent manner at a concentration as low as approx. 0.02% or 0.2 mg/ml (**Figure 1A**; **Table 1**). Interestingly, the immortalized mesothelial MeT5A cell line was substantially more resistant to QV0 treatment (1.2 ± 0.06 mg/ml) than any of the PM cell lines (mean IC50 was 0.1 ± 0.05 mg/ml; next highest IC50 was 0.2 ± 0.02 mg/ml) (**Table 1**). Furthermore, we found that the QV0-treated PM cells at IC50 showed a significantly shorter migration distance in 24 h (Welch two-sample t-test, p = 0.003, **Figures 1B, C**; **Supplementary Data 1**) and suppressed the colony formation of cancer cells in 14 days (**Figure 1D**; **Supplementary Data 2**) (representative data of cells H2452 and MM05 are shown). Migration inhibition was further supported by live cell imaging on representative mesothelioma cells (H2452 and H28). Our results indicated that cells treated with QV0 are not mobile when compared to control cells (Supplementary video). However, in comparison to the normal cells, the treatment with QV0 for 48 h did not induce any alterations to the cell cycle phases in the cancer cells (**Figure 1E**). Collectively, these findings suggest that QV0 can inhibit cancer cell proliferation, cell migration, and colony formation.

**Table 1:** IC_50_ values (concentration at which 50% of cells are viable) for each cell line treated with QV0.

Cell line	IC_50_ (mg/ml)
MeT5A	1.234 ± 0.065
H28	0.170 ± 0.017
H226	0.100 ± 0.001
H2452	0.162 ± 0.001
MSTO	0.173 ± 0.006
REN	0.079 ± 0.0002
MM05	0.184 ± 0.002
VMC40	0.208 ± 0.019
AC29	0.082 ± 0.004

**Figure 2:**
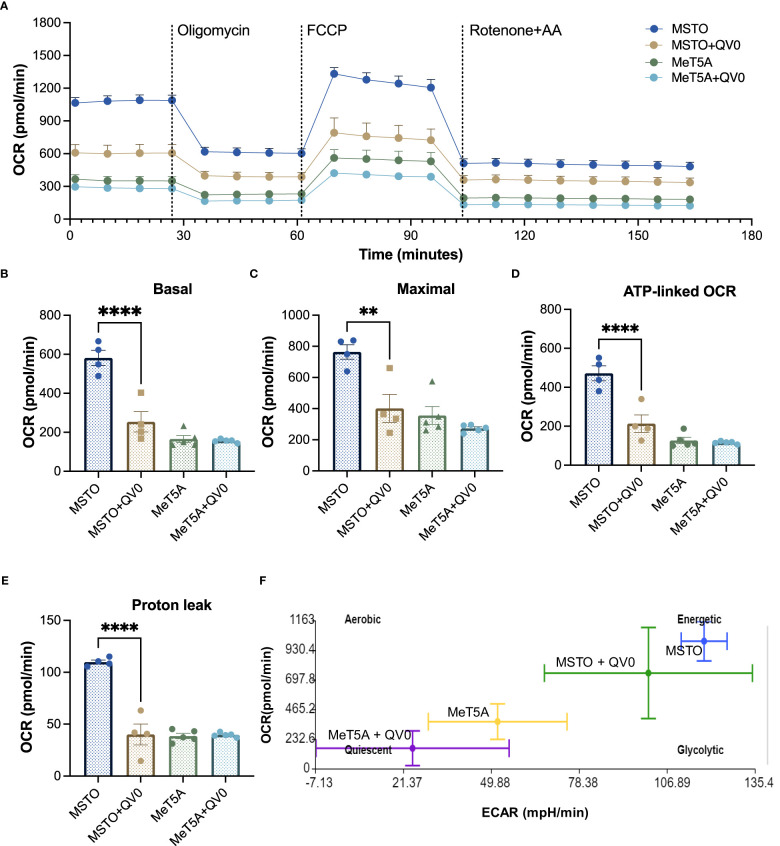
QV0 decreased PM cell energy demand and mitochondrial activity. **(A)** A representative profile of the mitochondrial stress test. Dotted vertical lines indicate the addition of 1 μM oligomycin, 0.5 μM FCCP, and 0.5 μM rotenone and antimycin **(A)** Graphs depicting **(B)** basal mitochondrial OCR, **(C)** maximal mitochondrial OCR, **(D)** ATP-linked OCR, **(E)** proton leak, and **(F)** mitochondrial respiratory energy map (ratio of OCR to ECAR). N = 5 per group, **P<0.01; ****P<0.0001.

We studied the effects of QV0 on cellular respiration and energy production in both non-cancer and PM cells. The mitochondrial respiratory profiles of immortalized mesothelial, MeT5A, and a well-established human PM cell line in our lab, MSTO, were analyzed using the Seahorse XF24 system following treatment with QV0. Live cells were sequentially injected with different mitochondrial respiration modulators, including oligomycin, phenylhydrazone (FCCP), rotenone, and antimycin (**Figure 2A**). Basal respiration measures the energetic demand of cells under basal conditions (**Figure 2B**), and maximal respiration represents the maximum capacity that the electrorespiratory chain can achieve following the injection of FCCP (**Figure 2C**). Adenosine triphosphate (ATP)-linked respiration is reflected by the decrease in oxygen consumption rate (OCR) following the injection of the ATP synthase inhibitor, oligomycin, which is the portion of basal respiration (**Figure 2D**). The remaining basal respiration not coupled to ATP synthesis after oligomycin injection represents proton leak (**Figure 2E**) (28).

Our results indicate that MSTO cells exhibited a higher level of mitochondrial activity with significantly higher basal, maximal, and ATP-linked respiration when compared to that of the immortalized mesothelial control, MeT5A (**Figure 2A**). Importantly, the treatment of QV0 significantly repressed the mitochondrial activity in MSTO cells, including basal (**Figure 2B**), maximal (**Figure 2C**), and ATP-linked OCR (**Figure 2D**) as early as 24 h post-QV0 treatment. Moreover, proton leakage, which can be a sign of mitochondrial damage, was significantly reduced in QV0-treated MSTO cells at 24 and 48 h, suggesting impaired mitochondrial function (**Figure 2E**). However, it was of interest that QV0 exerted a minimal effect on mitochondrial function in the immortalized mesothelial cell, MeT5A (**Figures 2B–E**). The basal extracellular acidification rate (ECAR) was plotted against OCR in **Figure 2F**, where the energetic (MSTO) and quiescent (MeT5A) bioenergetic profiles were demonstrated. A shift in bioenergetics was observed for MSTO following QV0 treatment after 24 h, with cells becoming less energetic and more quiescent as the ATP production pathways were inhibited.

**Figure 3:**
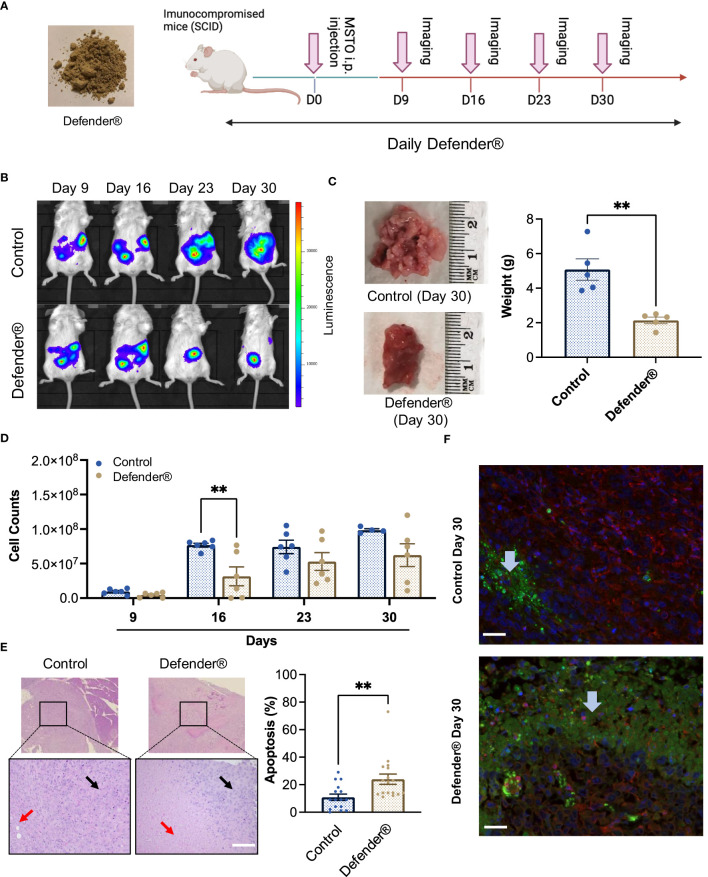
**(A)** SCID mice were inoculated with MSTO mesothelioma cells *via* intraperitoneal (i.p.) injection, followed by subsequent treatment with Defender^®^ or saline administered orally for 30 days. Pink arrows indicate the IVIS imager analysis schedule. **(B)** Representative images of animals treated with Defender^®^ show suppression of tumor growth with respect to the untreated control. **(C)** Defender^®^ treatment significantly reduced tumor size and weight at harvesting with respect to the untreated control. **(D)** Tumor growth was quantified by total cell counts as measured by the IVIS imaging system, which showed a reduction of tumor growth in animals treated with Defender^®^ with respect to the untreated control. **(E)** Representative images showing an extensive area of dead cells (red arrows) in the Defender^®^-treated tumor H&E sections compared to the untreated control. Black arrows indicate the area of live cancer cells. **(F)** TUNEL staining showed enhanced apoptosis in Defender^®^-treated tumors. Green, red, and blue staining correspond to apoptotic cells (TUNNEL mix), live cells (beta-actin), and nuclear DNA (DAPI), respectively. The blue arrow indicates an apoptosis area. N = 10 per group, **P<0.01.

We next investigated the *in vivo* antitumor effect of QV0 in a xenografted mesothelioma (MSTO) mouse model. The mice were fed daily with a dietary formulation of QV0, Defender®, at 0.25 mg/g/day for 30 days (**Figure 3A**). Tumor growth was indicated as cell counts and monitored using an IVIS imaging system following 9, 16, 23, and 30 days post-tumor implantation, respectively (**Figure 3B**). We found that the tumor volume increased progressively in control animals, with approximately 7.67e07 tumor cells measured at day 16 after tumor implantation. In comparison, mice treated with Defender® exhibited a significant reduction in tumor volume, with approx. 3.16e07 cells measured at day 16, representing a 41% inhibition of tumor growth with respect to the untreated control mice (**Figures 3B–D**). We continued the treatment beyond day 16 and monitored tumor growth in both groups of animals. At day 30, the size and weight of the tumors harvested from Defender®-treated mice were significantly reduced when compared to the untreated control mice (**Figure 3C**). Additionally, we observed the appearance of an extensive area of dead cells in the Defender®-treated tumor tissue, comprising approximately 23.94% of the tumor, which was notably higher than the 10.81% measured for the untreated control mice (**Figure 3E**). The application of terminal deoxynucleotidyl transferase dUTP nick end labeling (TUNEL) staining with beta-actin further supported this finding, with a higher level of apoptotic cells present in the Defender®-treated tumor sections compared to the untreated sections (**Figure 3F**). These results collectively show that Defender® containing QV0 has potent anti-cancer activity in the suppression of tumor growth in a PM animal model.

**Figure 4:**
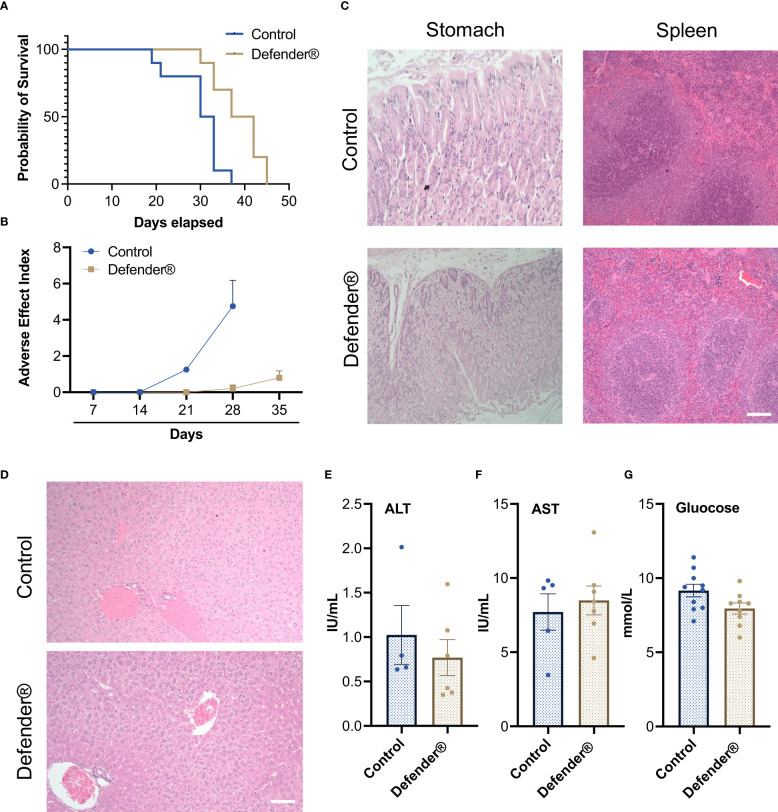
**(A)** Defender^®^ treatment demonstrated an increased survival rate with an extended median survival. **(B)** Defender^®^ treatment improved the adverse effect index after tumor bearing. **(C)** H&E staining showed that there were no major histological changes in stomach and spleen sections after Defender^®^ treatment. **(D)** There was no architectural distortion, cholestasis, ballooning, or fatty change in the liver of Defender^®^-treated animals. **(E–G)** No differences in serum ALT, AST, or glucose concentration were observed post-Defender^®^ treatment for 30 days. N = 10 per group.

We also observed a significant increase in survival rate in tumor-bearing mice treated with Defender^®^, which was on average 7 days longer than untreated control mice (**Figure 4A**). Additionally, we found that Defender^®^-treated mice had a reduced adverse effect index compared to the untreated control mice, which is measured based on criteria including a reduction in body weight, food intake, mobility, and the development of bleeding or diarrhea (**Figure 4B**). More importantly, the Defender^®^ administration did not induce a long-term systemic adverse effect in mice. For instance, the histological assessment of the stomach (gastric mucosa) showed no tumor involvement and no inflammatory features in both control and Defender^®^-treated animals. The spleens of control and Defender^®^-treated animals showed diffuse involvement from tumors dispersed as single cells. This resulted in a degree of disruption of the white and red pulp, but the architecture was preserved and discernible (**Figure 4C**). Tumor infiltration was observed in mice’s livers as small solid tumor nodules either with (20%) or without (66.7%) the treatment of Defender^®^; however, architectural distortion, cholestasis, ballooning, or fatty change that indicates liver toxicity was not detected (**Figure 4D**). Serum levels of alanine aminotransferase (ALT), aspartate aminotransferase (AST), and glucose were not changed after 30 days of the Defender^®^ treatment (**Figures 4E–G**), which collectively demonstrated that the oral administration of Defender^®^ for 30 days did not induce liver toxicity in the PM animal model.

**Figure 5:**
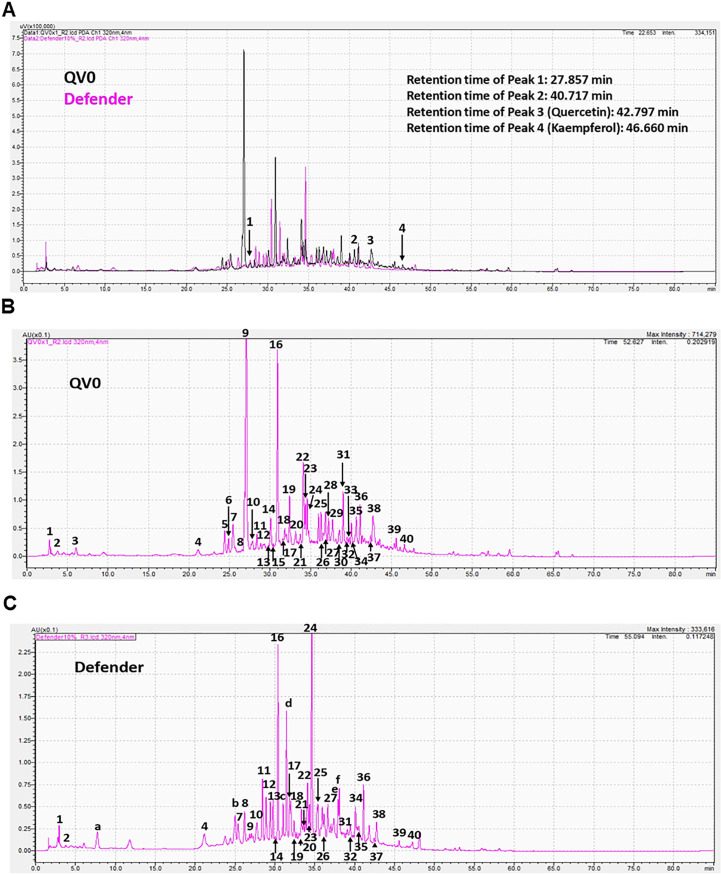
Chromatograms of *Leptospermum* extract (QV0) and Defender **(A)**; QV0 **(B)**; and Defender **(C)** measured at 320 nm using a photodiode array (PDA) detector.

To gain an understanding of the bioactive constituents of the *Leptospermum* extract, QV0, and its dietary formula, Defender^®^, we examined the chemical and antioxidant properties, specifically the total phenolic content and ferric antioxidant power (FRAP). The results demonstrated that Defender^®^ is a rich source of phenolic compounds, which are higher than those of QV0. Noticeably, the ferric antioxidant power of QV0 is significantly higher than that of Defender^®^ (**Table 2**). Quercetin and kaempferol are two phenolic compounds identified, and QV0 has significantly higher levels of these compounds as compared to Defender^®^. Of note, there are two compounds (peaks 1 and 2, **Figure 5**) that have been identified in QV0 and Defender^®^. QV0 has the highest levels of the two unknown compounds, followed by Defender^®^. In addition, the scanning results (**Figure 5**) revealed that there are over 30 major peaks that can be observed in both QV0 and Defender^®^, meaning there are over 30 major individual phytochemicals, and most of these compounds have not been identified.

**Table 2:** Total phenolic content, antioxidant activity, and some phytochemicals in *Leptospermum* extract (QV0) and Defender^®^.

	*Leptospermum* extract (QV0)	Defender^®^
TPC (mgGAE/g)	187.9 ± 40.7^a^	462.45 ± 59.61^b^
FRAP (mgTE/g)	320.7 ± 58.7 ^a^	156.25 ± 12.02 ^b^
Quercetin (μg/g)	2584.18 ± 20.53 ^a^	87.84 ± 2.78 ^b^
Kaempferol (μg/g)	460.84 ± 11.49 ^a^	6.67 ± 1.52 ^b^
Peak 1 (μgQE/g)	884.08 ± 130.31 ^a^	96.94 ± 9.01 ^b^
Peak 2 (μgQE/g)	2,060.87 ± 354.45 ^a^	74.56 ± 1.97 ^b^

*Values represent the mean ± standard deviation. Data in the same row not sharing similar superscript letters are not significantly different at p<0.05.

## Discussion

Chemotherapy is one of the most commonly administered treatments for mesothelioma; however, there are many adverse side effects associated with its use in PM patients (29). The current combination of ipilimumab and nivolumab immunotherapy has a similar response rate to chemotherapy, but the risk of immune-mediated adverse events is modest. The discovery of improved treatment options with minimal side effects is urgently needed. Natural extracts have continuously proven to be an important and rich source of anti-cancer therapies (30); however, limited studies have investigated their potential utility in the treatment of mesothelioma. In the present study, we report for the first time the anti-cancer effect of *Leptospermum* extract (QV0) on PM cells, as well as demonstrate that the food formula of QV0 (Defender^®^) can suppress mesothelioma tumor growth in a preclinical mouse model.

We investigated the anti-cancer activity of QV0 in PM cells, and our results indicated that QV0 can suppress cell proliferation and migration in eight tested mesothelioma cells (H28, MSTO, VMC40, H226, H2452, REN, MMO5, and AC29). Live cell image videos showed that QV0 treatment inhibited mesothelioma cell division and mobility, which was further proved by a migration assay. A clonogenic assay also indicated that QV0 significantly suppressed mesothelioma cell colony formation. Interestingly, the IC_50_ data indicated that an immobilized mesothelial cell, MeT5a, was less sensitive to the QV0 treatment at the effective concentration (0.2 g per 100 ml), whereas all cancer cells were found to be sensitive at QV0 concentrations ranging from 0.01 to 0.02 g per 100 ml. We concluded that QV0 has no significant toxicity to non-cancer cells.

In the present study, QV0 induced apoptosis both in the MSTO cell line (**Supplementary Data 3**) and in an animal model. The mechanisms of apoptosis are highly complex but are commonly caused by two main pathways: the extrinsic death receptor pathway and the intrinsic mitochondrial pathway. In the present study, QV0 treatment did not trigger cell cycle arrest, while previous studies demonstrated the ability of *Leptospermum* extracts to induce apoptosis. This can be explained by the different species in our study (*L. polygalifolium*) compared to previous studies (31, 32) (*Leptospermum javanicum*). In addition, QV0 is an aqueous extract, whereas previous studies tested LF1 (32), which was precipitated from ethanol extraction, and betulinic acid, which was further purified from LF1. Thus, the tested extracts are different. Since our results suggest apoptosis is not caused by cell cycle profile changes, we then performed the mitochondrial stress test to measure mitochondrial function, specifically mitochondrial OCR. The results demonstrated a significant reduction in the basal, maximal, and ATP-linked OCR in the QV0-treated cancer cells, with an evident shift in metabolic potential from energetic to quiescent as early as 24 h post-treatment. The suppression of proton leaks also indicated mitochondrial damage after QV0 treatment. These findings suggest that QV0 inhibits the mitochondrial OCR in mesothelioma cells, causing mitochondrial dysfunction-induced apoptosis. Our findings are in agreement with those of a study by Amran et al., which demonstrated that Tualang honey inhibits cell proliferation and induces cell apoptosis with reduced mitochondrial membrane potential in the human breast cancer cell lines MCF-7 and MDA-MB-231 (33). Moreover, we found that the anti-cancer effects of QV0 on proliferation, apoptosis, and mitochondrial function were cancer cell-specific, with QV0 having no effect on non-malignant cells (**Figure 1**), thus suggesting its potential to be used as a novel anti-cancer drug that induces minimal damage or alteration to healthy non-malignant cells. Overall, these comprehensive *in vitro* studies conclude that, as a natural plant extract, QV0 possesses desirable anti-cancer properties that are associated with and/or mediated by mitochondrial dysfunction-related apoptosis.

In a mesothelioma preclinical animal model, we established that Defender^®^-treated animals showed significant tumor suppression. Defender^®^-treated animals showed a reduction in tumor volume and an improved health index, which were associated with an average extended life expectancy of up to 7 days when compared to untreated animals. During the 30-day treatment period involving oral administration of Defender^®^, the animals showed no evident signs of liver toxicity, nor was there an increase in their blood glucose level. Additionally, the histology of the spleen, liver, and stomach post-Defender^®^ treatment was assessed by a pathologist, and no adverse side effects were observed. This finding does not reflect that of manufactured medicines, such as chemotherapy drugs, which typically induce multiple adverse side effects. Plant extracts such as those used in Traditional Chinese Medicine (TCM) have been practiced and developed over thousands of years (34); however, some have been known to cause liver toxicity (35). Given that our study showed no evident signs of liver toxicity following Defender^®^ treatment, this suggests that the potential use of Defender^®^ for the treatment of PM would be a safer alternative to conventional TCM and provides rationale for further testing of Defender^®^ in prospective human clinical trial studies. Furthermore, our results indicated that in Defender^®^-treated animals, the harvested tumors appeared to have a significant increase in cell death when compared to the untreated control animals. The tunnel assay confirmed that QV0 treatment induced mesothelioma cell apoptosis. This finding is concordant with similar studies by Navanesan et al., who demonstrated that *Leptospermum* subsp. (similar species of QV0), *javanicum* and *flavescens* are capable of inducing cell apoptosis and suppressing the metastatic potential of human lung carcinoma cells (31, 32).

The anti-cancer properties of QV0 and Defender^®^ can be attributed to their high levels of polyphenols, which possess strong antioxidant activity. Levels of polyphenols in QV0 and Defender^®^ are higher than those in ginseng root extract (36) and selected Chinese and Mexican medicinal plant extracts (37, 38). Although QV0 contains less than 50% polyphenols compared to that of the Defender^®^, the antioxidant activity of QV0 is significantly higher (double) than that of the Defender^®^, revealing that phenolic compounds in QV0 exhibit potent antioxidant activity compared to those of the Defender^®^, which only contains 5% of QV0. Of note, there are over 30 major individual compounds observed in **Figure 5**, but only two compounds have been identified: quercetin and kaempferol. These compounds are known to induce cytotoxic effects on cancer cells through several mechanisms, such as apoptosis, cell cycle arrest at the G2/M phase, and downregulation of epithelial–mesenchymal transition (EMT)-related markers (39, 40). Over 28 individual compounds are yet to be identified and tested for their anti-cancer properties. Therefore, future studies are warranted to isolate and characterize these compounds and to subsequently investigate their potential anti-cancer properties. Of note, there are two common peaks (peaks 1 and 2, **Figure 5**) that were observed in QV0 and Defender^®^. It is likely that these peaks correspond to the key bioactive compounds that are associated with the anti-cancer properties of QV0 and Defender^®^. Prospective studies are recommended to identify these compounds and their associated anti-cancer properties.

## Summary

PM is an aggressive malignancy of the lung lining with limited effective treatment options. In the present study, we have shown for the first time the promising anti-cancer potential of the tree *L. polygalifolium*-derived natural products, QV0, and Defender^®^. Specifically, this study demonstrates that QV0 exerts an inhibitory effect on PM tumor cell growth and improves host survival in a PM mouse model. These exciting findings provide an essential foundation and rationale for early-stage clinical trials, and we believe that prospective translational research will facilitate the successful implementation of QV0 in the clinical setting as a novel treatment option that will ultimately benefit PM patients.

## Data availability statement

The original contributions presented in the study are included in the article/**Supplementary Material**. Further inquiries can be directed to the corresponding author.

## Ethics statement

The animal study was reviewed and approved by the Sydney Local Health District.

## Author contributions

HS and YC conceived the project, conducted the experiments, and prepared the manuscript. LeZ, T-KY, LiZ, HNTP, and QV assisted in the experiments. ER and HS performed the data analysis. KL and SoK prepared sections and performed histology assessment, LeZ prepared figures, LeZ, HK, BJ, and StK edited manuscript. All authors contributed to the article and approved the submitted version.
