# Supplementary figures and images for: Leptospermum extract (QV0) suppresses pleural mesothelioma tumor growth in vitro and in vivo by mitochondrial dysfunction associated apoptosis
Source: Front Oncol. 2023 Jul 5;13:1162027. doi: 10.3389/fonc.2023.1162027 (PMC10354640; doi:10.3389/fonc.2023.1162027)

## Slide 1
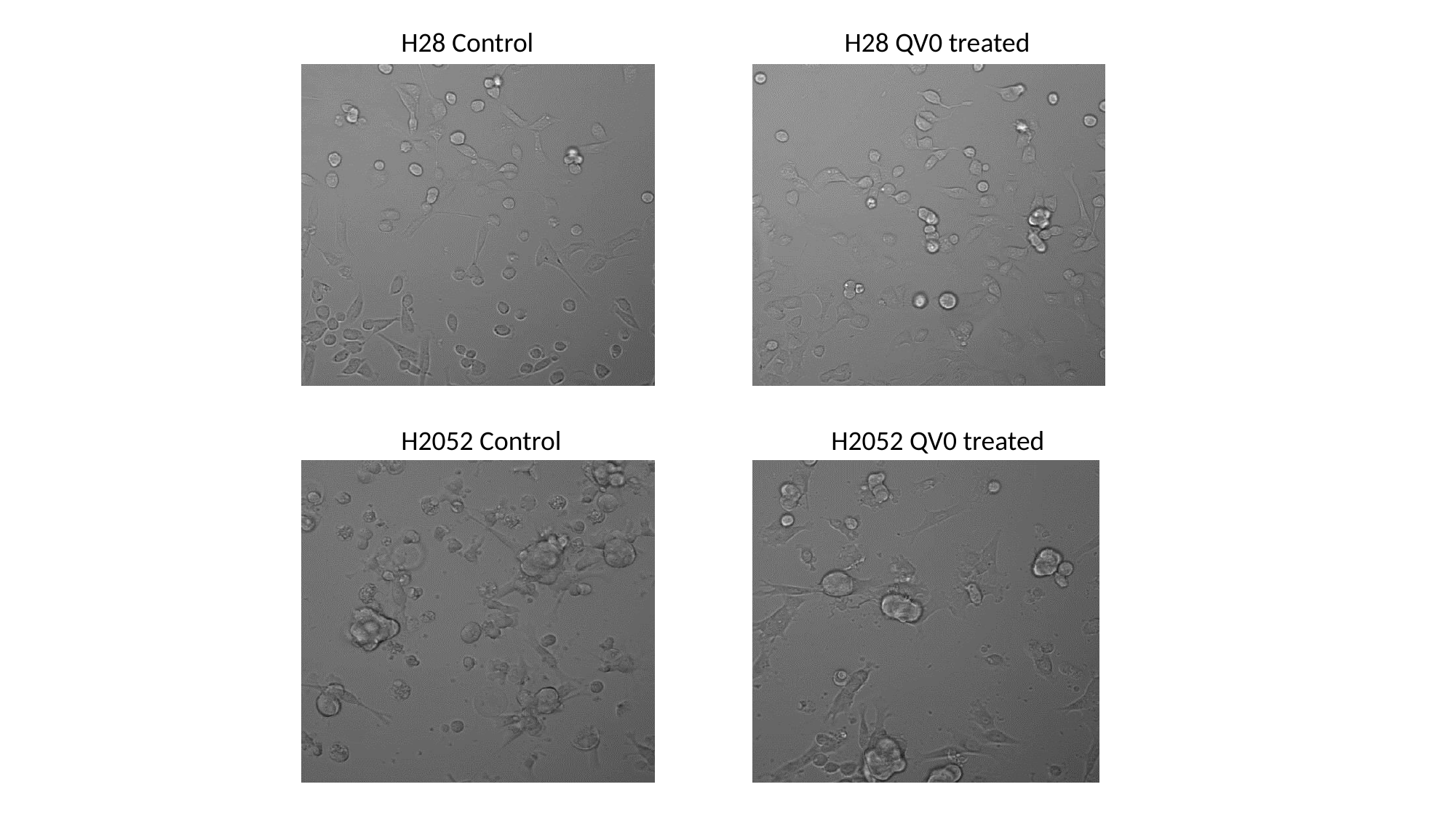

H28 Control
H28 QV0 treated
H2052 Control
H2052 QV0 treated

Supplement: Supplementary file 1 [file Presentation_1.pptx]
