# Supplementary material for: Leptospermum extract (QV0) suppresses pleural mesothelioma tumor growth in vitro and in vivo by mitochondrial dysfunction associated apoptosis
Source: Front Oncol. 2023 Jul 5;13:1162027. doi: 10.3389/fonc.2023.1162027 (PMC10354640; doi:10.3389/fonc.2023.1162027)
Supplement: Supplementary file 2 [file Table_1.docx]

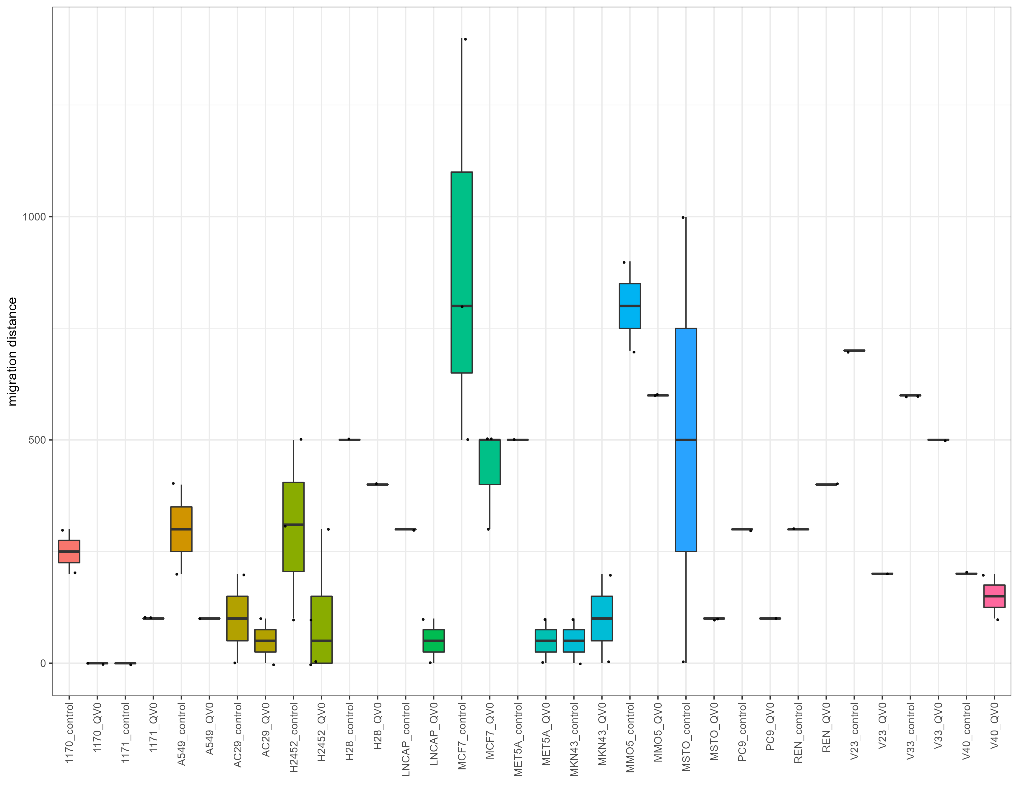
Supplementary data 1. Migration distance in all cell line tested. There is a statistically significant difference in migration distance between QV0-treated cells and controls (p-value=0.0002, mean of controls=48.1, mean of QV0-treated=21.0, 95% CI for difference in means= (13.5,40.7)).


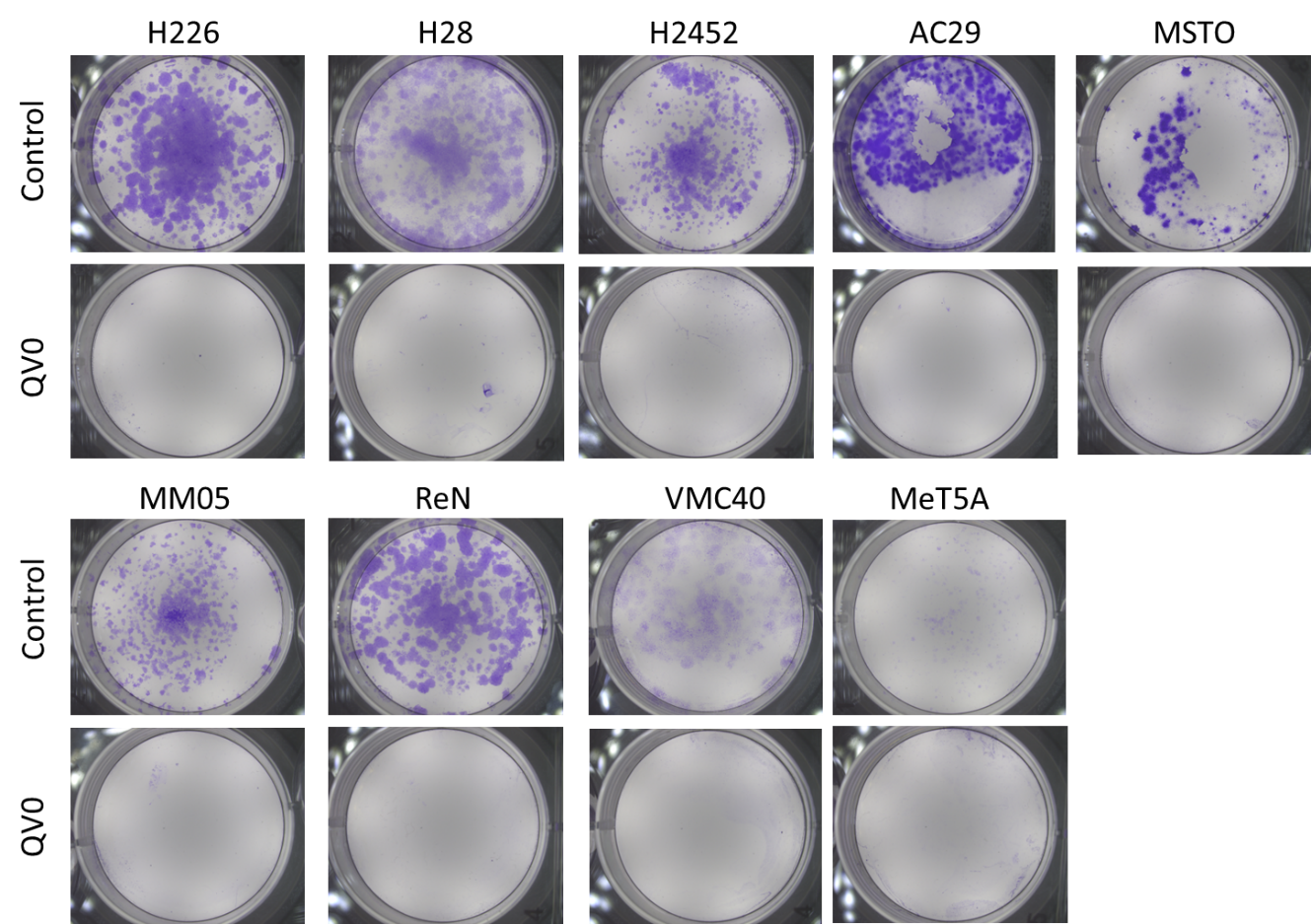


Supplementary data 2. Colony formation assay in eight mesothelioma cell lines and one immortalised mesothelial cell MeT5A.


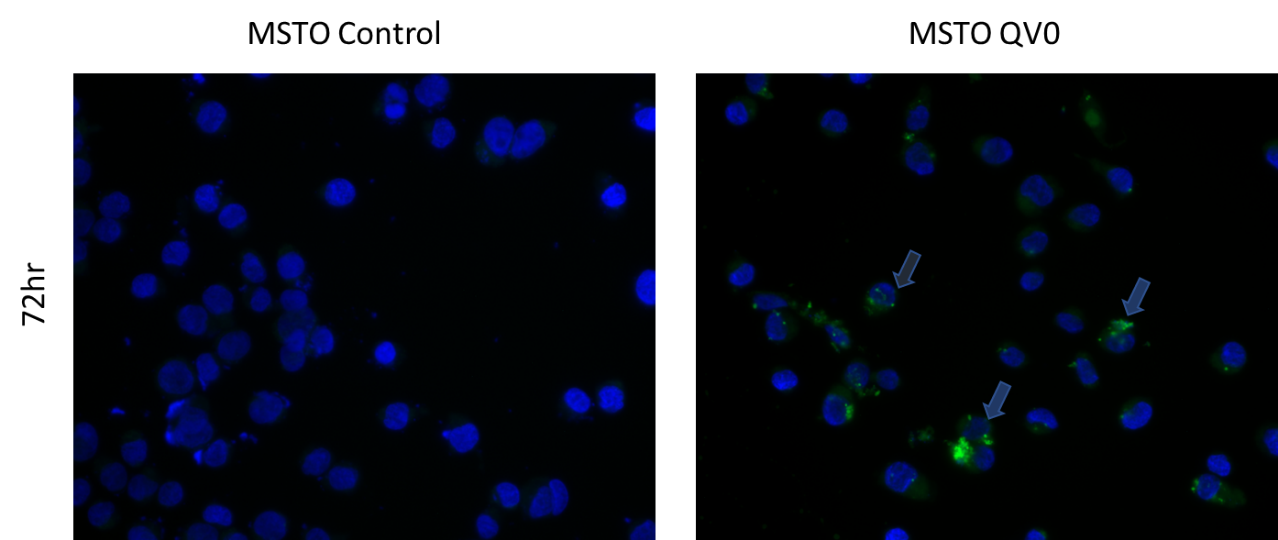


Supplementary data 3. QV0-induced apoptosis in MSTO mesothelioma cell line after 72 hr treatment. Cells are stained with *In Situ Cell Death Detection Kit*. Arrows indicate the cells under apoptosis.
